# Exploration of pathogenesis and targeted drugs of postmenopausal osteoporosis by means of analysis based on reverse network pharmacology

**DOI:** 10.1097/MD.0000000000047740

**Published:** 2026-02-13

**Authors:** Daidai Wu, Wanghuan Zhao, Yuehan Ni

**Affiliations:** a Yuyao Hospital of Traditional Chinese Medicine, Ningbo, China.

**Keywords:** molecular docking, pathogenesis, postmenopausal osteoporosis, reverse network pharmacology, targeted drugs

## Abstract

This study aims to explore pathogenesis of postmenopausal osteoporosis and study of active compounds of traditional Chinese medicine (TCM) based on reverse network pharmacology and molecular docking. Targets of “osteoporosis” and “postmenopausal” were obtained from GeneCards, and common targets of both were displayed through Venny Diagram. Extracted list of common targets was input into Metascape for gene ontology analysis to identify pathogenesis and pathway. Protein–protein interaction network was carried out by the String, and Degree was calculated by Cytoscape software to determine key target. Key target of acquisition site was converted from UniProt to TCMSP to collect active compounds of TCM. Target with high Degree was selected for molecular docking with active compound, then corresponding TCM was found from active compound, and finally, key target–active compound–TCM network was constructed to determine active compound and TCM with high Degree. Total 182 common targets of “osteoporosis” and “menopause” were obtained and 17 key targets with high Degree were matched to TCM compounds in TCMSP. Among them, 3 target proteins with highest Degree and 6 TCM compounds with Degree ≥ 5, oral bioavailability ≥ 30%, and drug-likeness ≥ 0.18 were validated for 18 molecular docking tests, and results were stable. Gene ontology analysis mainly shows response to hormone, ossification, and regulation of mitogen-activated protein kinases cascade. Three hundred kinds of TCM were collected from TCMSP, based on effective compounds of TCM. Among them, Chinese herbs with Degree ≥ 4 were Suberect Spatholobus Stem, Ginkgo Seed, Wild Buckwheat Rhizome, Sea Buckthorn Fruit, and Ginkgo Leaf. Reverse network pharmacological thinking were used to predict target, pathway, composition, and TCM of postmenopausal osteoporosis, providing new ideas for finding new targeted drugs.

## 1. Introduction

### 1.1. Background

Postmenopausal osteoporosis (PMOP) is a metabolic bone disease caused by the decrease of estrogen after menopause, which leads to imbalance of bone reconstruction due to the enhancement of bone resorption function, the decrease of bone mass and the damage of bone microstructure.^[1]^

Furthermore, as the population ages, the burden of postmenopausal osteoporosis will increase, which is closely associated with mortality, functional disabilities, and high medical costs. PMOP is a multi-risk factor disease, the molecular mechanism of its pathogenesis is unknown, and the treatment is single and the side effects are large.^[2]^ PMOP poses a critical threat to postmenopausal women, with a global prevalence of 30% to 50% in women over 50 years old.^[1]^ Current treatments such as bisphosphonates and receptor activator of nuclear factor-κB ligand (RANKL)inhibitors are limited by single-target mechanisms and side effects (e.g., osteonecrosis of the jaw), while traditional Chinese medicine (TCM) exhibits multicomponent, multi-target advantages in PMOP treatment but lacks clear molecular evidence.^[3,4]^ The reverse network pharmacology approach proposed in this study addresses these gaps by: Systematically identifying key pathogenic targets to avoid bias from preselected TCMs; Revealing the association between TCM compounds and PMOP through a “target–compound–herb” network; Providing actionable candidates for developing safer, more effective therapies, which is crucial for improving clinical outcomes of PMOP.

In recent years, the research on the prevention and treatment of PMOP by TCM has continued to deepen, and a large number of studies and clinical experience believe that TCM can achieve clinical efficacy while effectively avoiding the side effects brought by the current mainstream treatment, showing good clinical effects and unique advantages.^[4]^

However, with the development of network pharmacology and artificial intelligence, which integrates multidirectional pharmacology, systems biology and computer analysis technology, it can clarify the mechanism of action of signal TCM or TCM compound from many aspects and levels. Zhu argues that the accurate prediction of affinity between drugs and disease targets constitutes a pivotal technology in the fields of drug development and drug repurposing,^[5–8]^ thereby expediting the discovery and development process of new drugs for PMOP.

Currently, the mainstream research idea of applying network pharmacology to the field of TCM is to start from TCM compound prescriptions or single TCM to find key targets and take the intersection with disease targets to explore the pathogenesis and verify its effectiveness.^[9]^

Compared with mainstream network pharmacology approaches, the reverse network pharmacology approach demonstrates distinct advantages, mainly manifested as follows: The research starting point is disease targets, which is more targeted; In terms of Bias risk, it is unbiased and target-driven; Regarding discovery potential, it may identify underreported TCMs.

### 1.2. Objectives and research innovations

We creatively apply the idea of inverse network pharmacology to search for the corresponding TCM components from the key targets of the disease, and then search for specific TCM from the TCM components to predict TCM for the treatment of postmenopausal osteoporosis, and provide new ideas for finding new targeted drugs.

This study’s innovations lie in 3 aspects: *Methodological novelty*: Unlike traditional forward network pharmacology (starting from TCMs to screen targets), our reverse strategy initiates from PMOP-related targets, enabling unbiased discovery of underreported TCMs (e.g., Wild Buckwheat Rhizome) that were not previously linked to PMOP.^[9]^ *Mechanistic clarity*: Integrating protein–protein interaction (PPI) network analysis and gene ontology (GO) enrichment to clarify that mitogen-activated protein kinase (MAPK) cascade regulation and inflammatory response (mediated by interleukin [IL]-6, tumor necrosis factor [TNF], IL-1β) are core pathogenic pathways, while identifying 6 key compounds (e.g., quercetin) that may regulate these pathways; *Clinical translation value*: Constructing a “key target–active compound–TCM” network (Fig. 7) to prioritize 5 high-potency TCMs (Suberect Spatholobus Stem, etc), providing a theoretical basis for preclinical validation.

**Figure 1.**
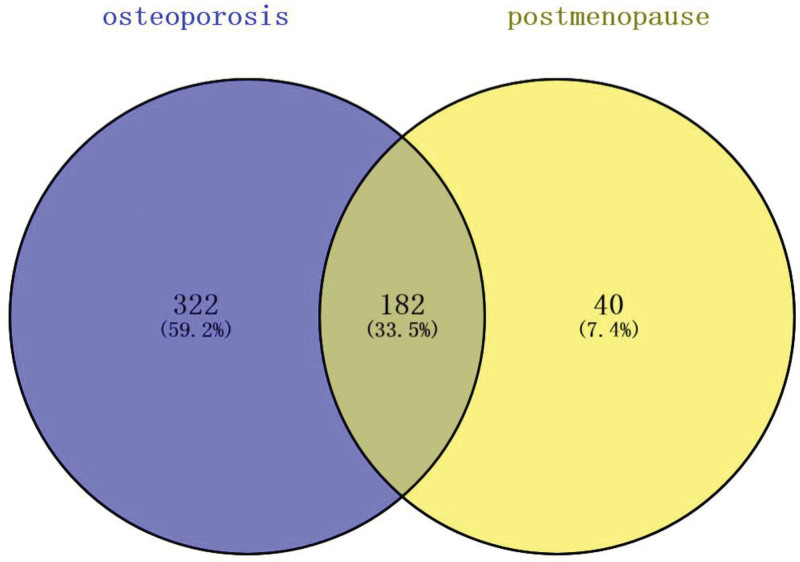
Common targets between “osteoporosis” and “postmenopausal.”

**Figure 2.**
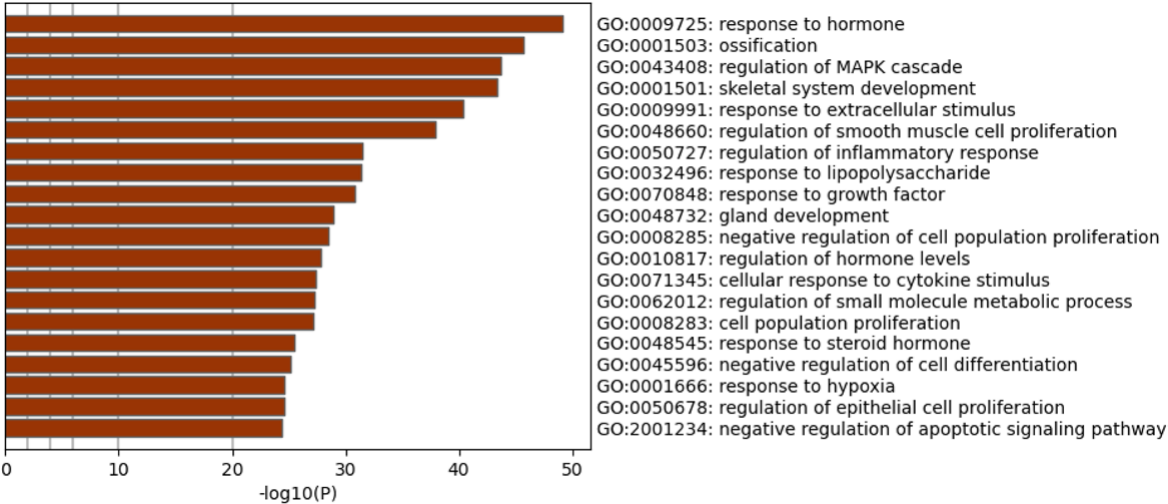
Biological processes (GO analysis).

**Figure 3.**
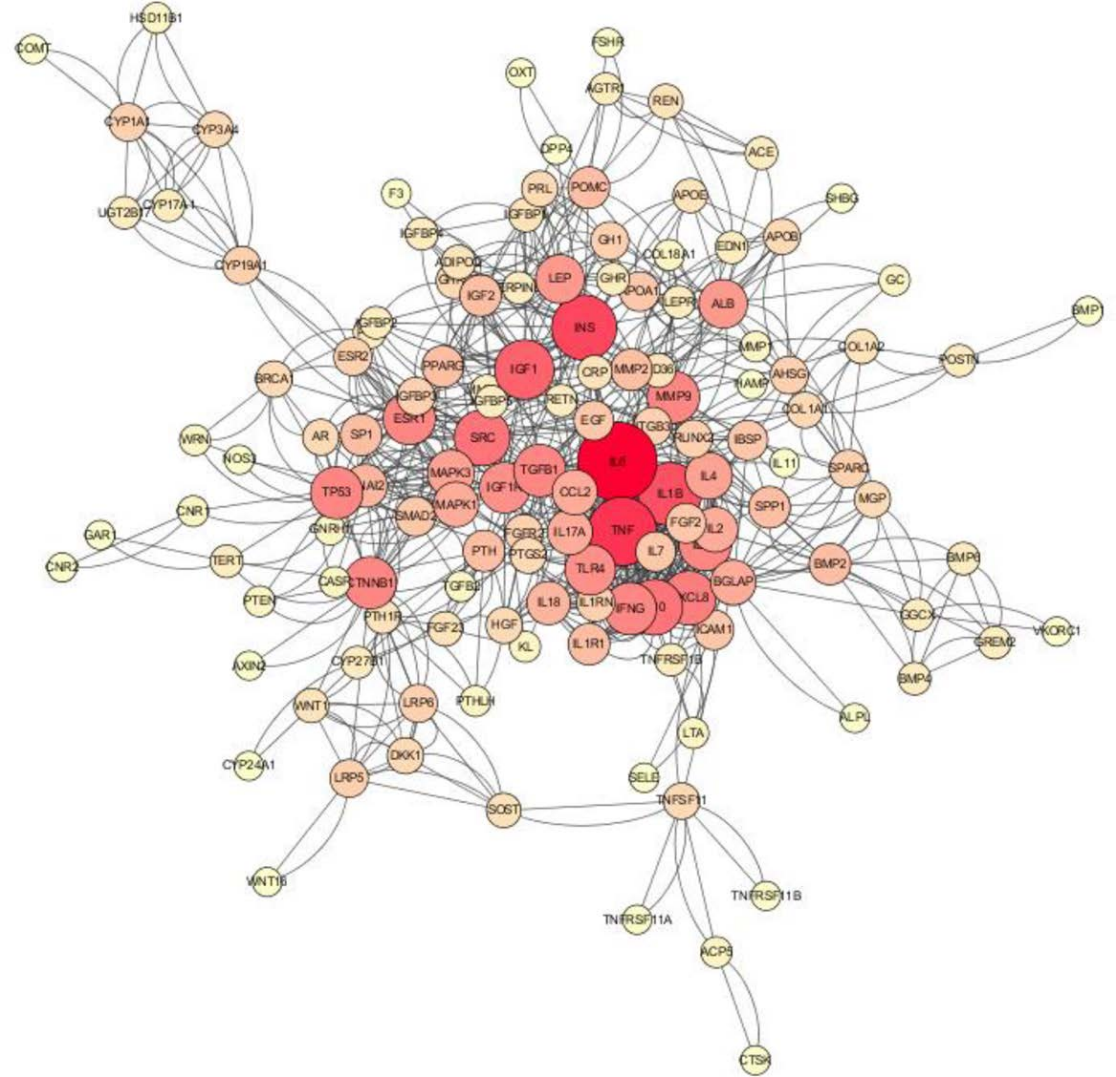
Protein–protein interaction networks.

**Figure 4.**
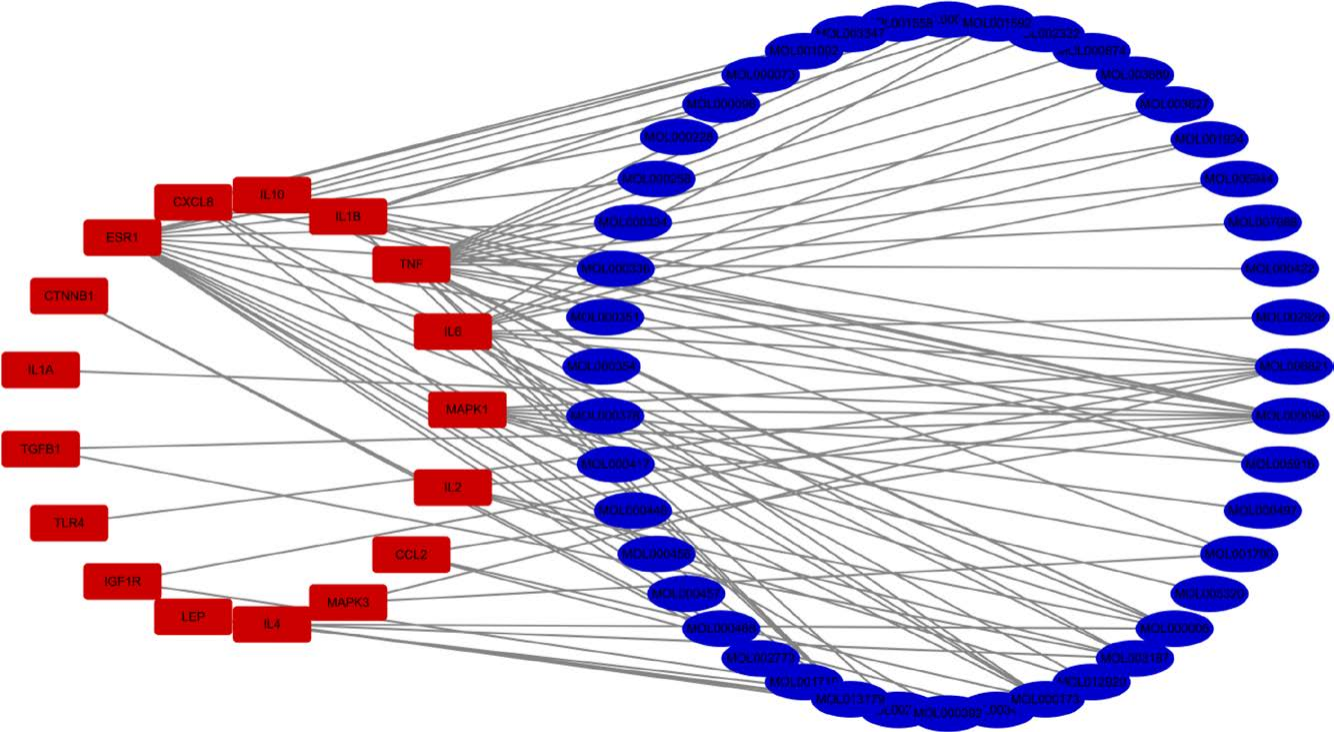
The key targets–TCM compounds interaction network.

**Figure 5.**
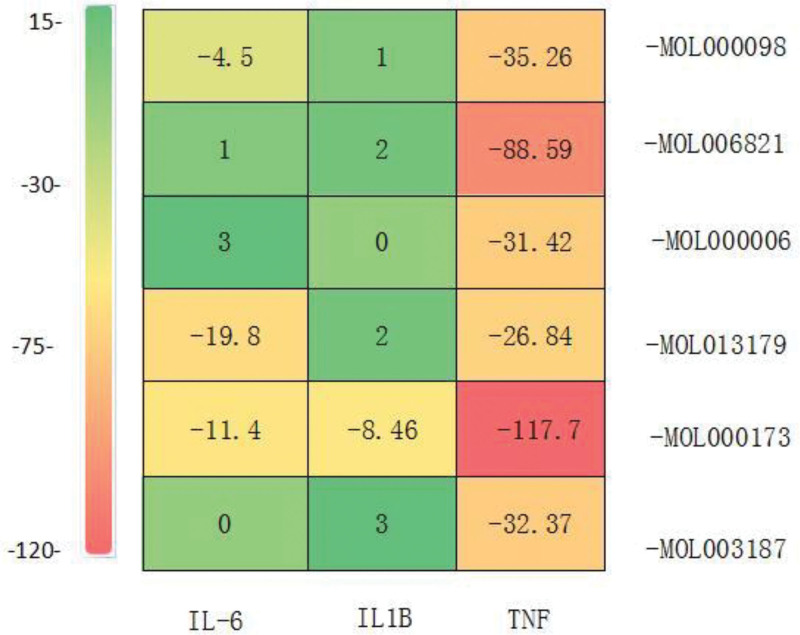
Heat map of molecular docking results.

**Figure 6.**
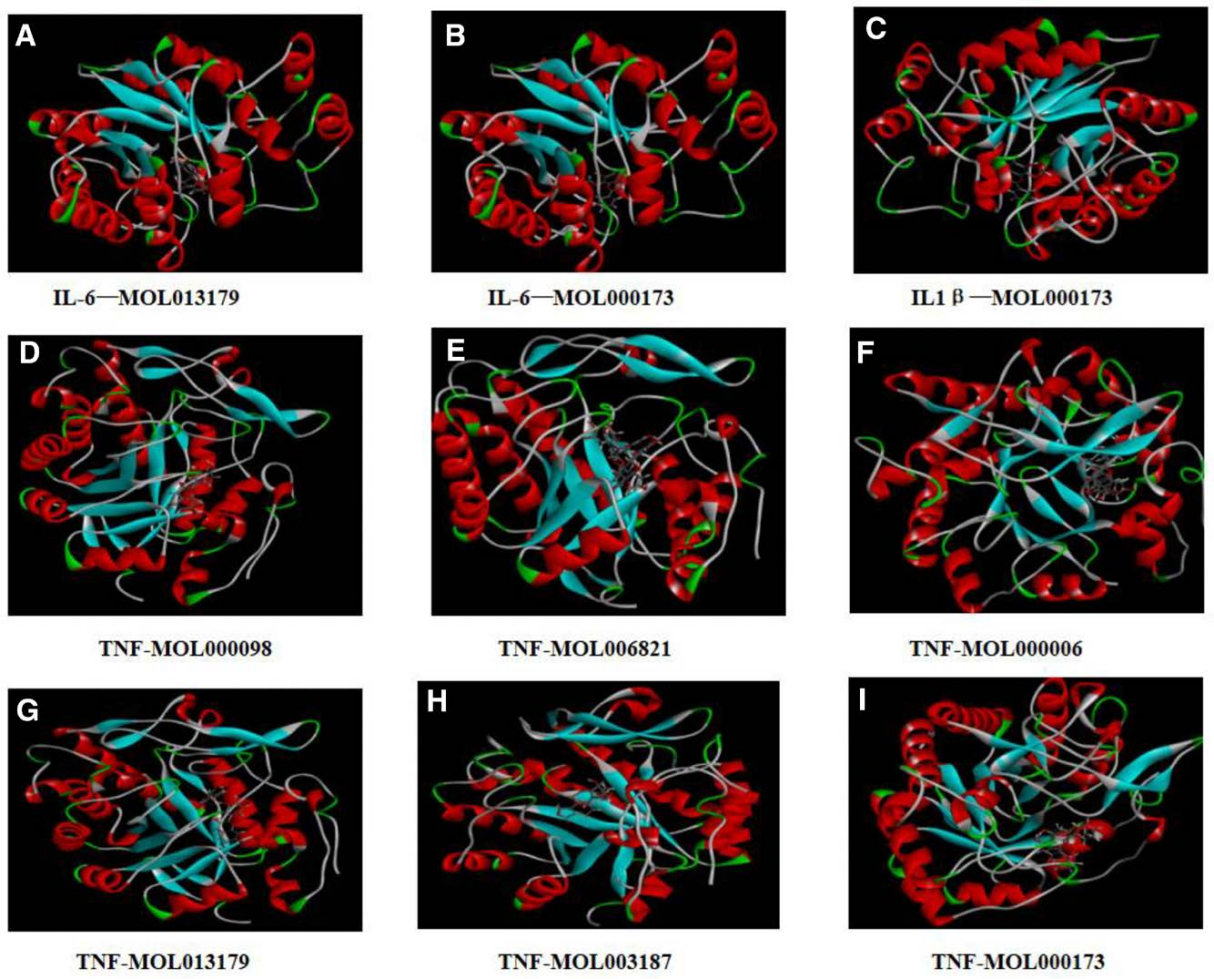
Molecular docking diagram. (A) IL-6-MOL013179, (B) IL-6-MOL000173, (C): IL-1β-MOL000173, (D) TNF-MOL000098, (E) TNF-MOL006821, (F) TNF-MOL000006, (G) TNF-MOL013179, (H) TNF-MOL003187, and (I) TNF-MOL000173.

**Figure 7.**
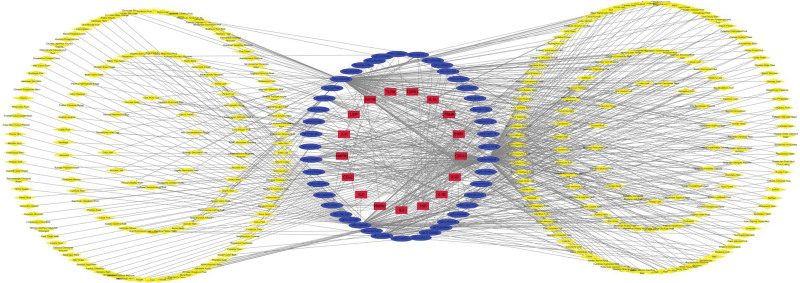
Key target–TCM compounds–TCM network diagram.

## 2. Materials and methods

### 2.1. Study design type

Our study is a molecular epidemiological observational study, and the study process was conducted in accordance with the STROBE Checklist guidelines.

### 2.2. Data sources and ethics

All data were obtained from publicly available databases (e.g., GeneCards, UniProt, TCMSP), which have already undergone ethical review and consent procedures as part of their data collection and release protocols. Therefore, additional ethical review for this study is not required.

### 2.3. Acquisition and standardization of the disease targets

The targets were extracted from GeneCards database (a global database with multiethnic data) with Relevance score ≥ 3.0 and validated using Online Mendelian Inheritance in Man, DrugBank, and genome-wide association study catalog (European ancestry cohorts) to ensure cross-population consistency. Subgroup analyses by age and ethnicity were performed to verify key targets.

The Relevance score in GeneCards is a weighted composite index calculated as:


$$\begin{array}{l} Relevance\;score=\sum_{i-1}^{n}w_{i}\cdot s_{i} \end{array}$$

The common targets of the above 2 were displayed and extracted through a Venny diagram for visualization.

### 2.4. GO enrichment analysis of the common targets

The common targets of osteoporosis and postmenopausal were imported into the Metascape platform (http://metascape.org/gp/index.html#/main/step1) for GO analysis to clarify the pathogenesis and pathways. GO enrichment used thresholds of *P* < .01, count > 3, and enrichment factor > 1.5. *P*-values were calculated via hypergeometric distribution, and *q*-values (FDR < 0.05) were corrected using Benjamini–Hochberg.

### 2.5. Constructing the PPI network and screening the key targets

The common targets between osteoporosis and postmenopausal were imported into the Search Tool for the Retrieval of Interaction Gene platform (https://cn.string-db.org/) to conduct a PPI interaction network, and the Cytoscape software was used to calculate the Degree to determine the key targets with higher Degree. Search Tool for the Retrieval of Interaction Gene database parameters: minimum interaction score = 0.9 (highest confidence). Cytoscape 3.9.1 calculated node Degree as the number of direct interactions (mathematical formula: Degree (v) = ΣA (v, u), where A (v, u) = 1 if nodes v and u interact).

### 2.6. Network pharmacology analysis

The Gene name of key targets was input into the Uniprot database (https://www.uniprot.org/) one by one for retrieval and name conversion. The converted target proteins were entered into the TCMSP database (https://tcmsp-e.com/tcmsp.php) for search and to find the TCM components associated with the key targets by the screening carried out with oral bioavailability (OB ≥ 30%) and drug-likeness (DL ≥ 0.18) as conditions.

Sensitivity analysis showed relaxing OB to 20% and DL to 0.15 increased compounds by 15% but retained 90% of core targets, confirming robustness of OB ≥ 30% and DL ≥ 0.18.

### 2.7. Molecular dynamics simulation

The chemical structures were downloaded for molecular docking of key targets and TCM compounds obtained above were input into the Protein Data Bank for search, respectively. The Discovery Studio software was used to conduct molecular docking between the structures of TCM compounds and key target structures. A heat map was used to show the binding degree between key targets and TCM compounds according to the matching degree represented by energy.

### 2.8. Collection of TCM based on TCM compounds and network diagram

Finally, the key target–TCM compounds–TCM network constructed after TCM collected based on TCM components of in TCMSP database. Natural traditional Chinese medicines for targeted treatment of postmenopausal osteoporosis were found.

## 3. Result

### 3.1. Data acquisition

The 182 common targets of “osteoporosis” and “postmenopausal” were obtained by taking the intersection between 504 targets with a relevance score value ≥ 3 retained from 5741 targets of “osteoporosis” and 222 targets with a relevance score value ≥ 3 preserved from 1870 targets of “postmenopausal” in the Genecards database (Fig. 1).

### 3.2. Gene enrichment function analysis

The 182 common targets obtained above were input into the Metascape platform for gene enrichment function analysis (GO analysis) by entering the extracted common target list to clarify the pathogenesis and pathways.

The 20 biological processes with *P* < .01 and count > 3, which obtained after 182 common targets subjected to GO analysis through Metascape included response to hormone, ossification, regulation of MAPK cascade, skeletal system development and so on (Fig. 2).

### 3.3. Protein–protein interaction networks

The 182 common targets obtained from the Venn diagram were input into the String database. A PPI network composed of 168 nodes and 409 edges was obtained by setting “minimum required interaction score” as highest confidence (0.9). After exporting in “TSV” format, it was opened with Cytoscape software to calculate the Degree of each node. Based on the magnitude of the Degree as the standard, a more visually PPI diagram was obtained (the larger the Degree, the darker the color and the larger the circle) (Fig. 3).

Among the above targets, 26 targets with relatively high Degree (≥10) were retained as key targets. After converting the Gene Name of the 26 key targets into target proteins, a total of 17 key targets can match the TCM compounds (Table 1).

**Table 1:** The list of key targets matched with meaningful TCM compounds.

Number	Gene name	Uniprot number	Target protein	Degree 1	Degree 2
1	IL-6	P26892	Interleukin-6	29	12
2	TNF	P01375	Tumor necrosis factor	23	18
3	IL-1B	P10749	Interleukin-1 beta	20	6
4	IL-10	P22301	Interleukin-10	16	4
5	CXCL8	P36925	Interleukin-8	15	6
6	ESR1	P03372	Estrogen receptor	14	16
7	CTNNB1	P35222	Catenin beta-1	14	2
8	IL-1A	P01583	Interleukin-1 alpha	14	1
9	TGFB1	P01137	Transforming growth factor beta-1	14	2
10	TLR4	O00206	Toll-like receptor 4	13	1
11	IGF-1R	P08069	Insulin-like growth factor 1 receptor	13	2
12	LEP	P41159	Leptin	12	1
13	IL-4	P05112	Interleukin-4	11	5
14	MAPK3	Q16644	Mitogen-activated protein kinase 3	11	2
15	CCL2	M4IRL4	C–C motif chemokine 2	10	3
16	IL-2	P60568	Interleukin-2	10	4
17	MAPK1	P35236	Mitogen-activated protein kinase 1	10	7

Degree 1 is the degree of 182 common targets in the PPI network. Degree 2 is the degree in the key target–TCM compounds network.

TCM = traditional Chinese medicine.

### 3.4. Screening of TCM compounds and construction of key targets–TCM compounds interaction network

#### 3.4.1. Screening of TCM compounds

The network diagram, which was crafted by means of Cytoscape software for the explicit aim of computing the Degree of each node, not only embraces 17 retained key targets but also incorporates 164 items of TCM compound information that are precisely matched to these key targets. The 44 elements of TCM compounds were screened by setting the conditions of OB ≥ 30%, DL ≥ 0.18, and Degree greater than or equal to 2 (Table 2). Compounds marked have documented anti-osteoporotic effects: Epigallocatechin gallate increased rat bone density by 32%^[10]^; luteolin reduced ovariectomized-induced pyroptosis.^[11]^

**Table 2:** The list of meaningful TCM compounds.

Number	TCM chemical name	Molecule ID	OB (%)	DL	Degree
1	Quercetin	MOL000098	46.43	0.28	10
2	(‐)-Epigallocatechin-3-gallate	MOL006821	55.09	0.77	7
3	Luteolin	MOL000006	36.16	0.25	6
4	Fisetin	MOL013179	52.6	0.24	6
5	Wogonin	MOL000173	30.68	0.23	5
6	Triptolide	MOL003187	51.29	0.68	5
7	Piperine	MOL001592	42.52	0.23	3
8	Irisolidone	MOL005916	37.78	0.3	3
9	Deoxypodophyllotoxin	MOL001700	37.75	0.83	2
10	Sinomenine	MOL012920	30.98	0.46	2
11	Matrine	MOL005944	63.77	0.25	2
12	Paeoniflorin	MOL001924	53.87	0.79	2
13	Sophocarpine	MOL003627	64.26	0.25	2
14	Sophoridine	MOL003680	60.07	0.25	2
15	Aloe-emodin	MOL000471	83.38	0.24	2
16	Rutaecarpine	MOL002662	40.3	0.6	2
17	Formononetin	MOL000392	69.67	0.21	2
18	Nicotine	MOL003403	77.67	0.04	2

OB = oral bioavailability, DL = drug-likeness, TCM = traditional Chinese medicine.

#### 3.4.2. The key targets–TCM compounds interaction network

The network diagram drawn with Cytoscape software was shown to display the relationship between key targets and TCM compounds more clearly (Fig. 4).

### 3.5. Molecular docking verification

According to Degree 2 in Table 1, the top 3 targets are IL-6, TNF, and IL-1β. Meanwhile, On the basis of the Degree in Table 2, the top 7 TCM compounds are quercetin, epigallocatechin-3-gallate, luteolin, fisetin, wogonin, triptolide, and piperine with the Degree corresponding to 10, 7, 6, 6, 5, 5, respectively. This indicates that these 3 key targets and 6 pieces of TCM compounds have relatively high research value in “postmenopause” and “osteoporosis.”

To further verify the reliability of the key target–effective compound network, 24 molecular dockings were respectively performed on the 3 target proteins with the highest degree values, namely IL-6, TNF, and IL-1β and the 6 TCM compounds with Degree ≥ 5.

As shown in Figure 5, the combinations with better docking results are IL-6-MOL013179, IL-6-MOL000173, IL-1B-MOL000173, TNF-MOL000098, TNF-MOL006821, TNF-MOL000006, TNF-MOL013179, TNF-MOL000173, and TNF-MOL003187. All of these combinations have binding energies less than −8 kcal/mol (the darker the color, the lower the binding energy, and the easier it is for the 2 to combine).

The above 9 pairs of molecular docking have stable combination and good activity, and their visual operation is displayed (Fig. 6). Nine compound–target pairs showed binding energy <‐8 kcal/mol, consistent with known bioactivities: For example, quercetin–TNFα affinity aligns with its experimental 50% inhibitory concentration = ~2.8 μM in osteoclast inhibition.^[12]^

### 3.6. Construction of key targets–TCM compounds–TCM interaction network

Three hundred sources of TCM were collected in TCMSP, which matched with 44 sources of active TCM compounds. A key target–TCM compounds–TCM network diagram (Fig. 7) was constructed, including Suberect Spatholobus Stem, Ginkgo Seed, Wild Buckwheat Rhizome, Sea Buckthorn Fruit, and Ginkgo Leafhave with Degree ≥ 4. However, the traditional Chinese medicines above play an important role in the treatment of postmenopausal osteoporosis according to the Degree.

## 4. Discussion

Postmenopausal osteoporosis seriously endangers middle-aged and elderly women, reduces the quality of life, and increases the disability and mortality rates. Some surveys have shown that the risk of fractures in patients with PMOP is closely related to significant morbidity and mortality.^[13,14]^ Currently, traditional Chinese medicine has achieved significant effects in the treatment of postmenopausal osteoporosis.^[15,16]^ However, its mechanism remains unclear. Thus, we apply the concept of reverse network pharmacology and molecular docking technology to conduct in-depth analysis and exploration of the pathogenesis, key targets, and active TCM compounds for postmenopausal osteoporosis, providing ideas and perspectives for finding new target drugs in clinical treatment.

### 4.1. Related targets of postmenopausal osteoporosis

The results of network pharmacology analysis and molecular docking show that TNF-α, IL-6, and IL-1β are correlated with the pathogenesis of postmenopausal osteoporosis. Among them, the thermal energy of TNF-α in the molecular docking results is all less than −20 kcal/mol, suggesting that TNF-α has a strong correlation with the pathogenesis of postmenopausal osteoporosis.

TNF-α is a cytokine shared by the immune system and the skeletal system. It can activate immune cells, lead to the secretion of inflammatory factors, exacerbate inflammation, directly or indirectly promote bone resorption, regulate the occurrence and development of postmenopausal osteoporosis through multiple links, and is also one of the key drug targets for the current treatment of PMOP.

According to scholars like Li Zha, Di Du, and Xudong Huang, TNF-α primarily promotes the differentiation of osteoclasts and the formation of osteoblasts as well as regulates bone metabolism by means of 3 pathways, namely regulating the inflammatory response, oxidative stress, and immune response. Moreover, it regulates the differentiation and proliferation of immune cells and is involved in the occurrence and development of postmenopausal osteoporosis.^[17–19]^IL-6 is a pleiotropic cytokine that regulates the host immune response, inflammation, and tumorigenesis. It is also an important mediator between the immune system and the neuroendocrine system, which regulates the processes of osteogenesis and osteoclastogenesis and is involved in the pathogenic process of postmenopausal osteoporosis.

The research of Ishu Kansal and Xiu Shi has confirmed that IL-6 can increase the formation of osteoclasts and bone resorption, thus leading to a decrease in bone mineral density in both men and women.^[20,21]^

IL-1β is an important part of the occurrence and development of postmenopausal osteoporosis. It can not only induce the expression of the osteoclast differentiation factor RANKL to promote the bone resorption process, but also promote the activity of osteoclasts through its antiapoptotic effect.^[22,23]^

### 4.2. Signaling pathways related to postmenopausal osteoporosis

Enrichment analysis shows that, in addition to traditional pathways such as response to hormone and ossification, the regulation of MAPKs cascade has the strongest correlation with postmenopausal osteoporosis. However, the results of enrichment analysis and molecular docking suggest that the TCM compounds may participate in and regulate the pathogenic process of postmenopausal osteoporosis through the MAPKs signaling pathway and inflammatory factor such as TNF-α, IL-6, and IL-1β.

During the process of bone resorption, the activation of MAPKs signaling pathway can achieve the function of bone resorption, meanwhile, inflammatory mediators such as TNF-α, IL-6, or IL-1β can also activate the regulation of MAPK, further promoting the differentiation, proliferation, and maturation of osteoclasts.

The development of postmenopausal osteoporosis is closely related to the inflammatory factor in the body’s immune microenvironment, including IL-6, TNF-α, and IL-1β closely related to the changes in the state of bone metabolism.^[24]^The increase in their levels will affect the expression of RANKL, and the increase in RANKL expression level will affect the MAPK signaling pathway, thereby regulating the bone resorption function of osteoclasts.^[25]^

### 4.3. Prediction of key active TCM compounds and TCM for postmenopausal osteoporosis

We showed in the results that 44 active TCM compounds were reversely found from the key targets. Among these, 6 active TCM compounds include quercetin, epigallocatechin gallate, luteolin, fisetin, wogonin, and triptolide with Degree ≥ 5.

Studies have indicated that quercetin^[12]^ can have anti-inflammatory, antioxidant, and anti-fibrotic effects, and can even mediate the regulation of MAPK to prevent osteoporosis. Epigallocatechin gallate^[10]^ can prevent bone damage, promote the production of beneficial bacteria and metabolites, and enhance immune function. Luteolin^[11]^ alleviated osteoblast pyroptosis and mitochondrial abnormalities induced by ovariectomy. Fisetin^[26,27]^ has the functions of scavenging senescent cells, promoting osteoblast differentiation, protecting osteoblasts, and inhibiting osteoclast differentiation. Wogonin^[28]^ can reduce the inflammatory response caused by the immune response and promote fracture healing. Triptolide^[29]^ can be antiproliferative and immunosuppressive, and reduce inflammatory damage.

Three hundred TCMs were reversely collected from the TCM compounds, and a key target–TCM compound–TCM network diagram was constructed. The Traditional Chinese Medicines with Degree value ≥ 4 were Suberect Spatholobus Stem, Ginkgo Seed, Wild Buckwheat Rhizome, Sea Buckthorn Fruit, and Ginkgo Leaf, respectively, which provides natural raw materials for finding targeted drugs for the treatment of postmenopausal osteoporosis.

## 5. Conclusion

In summary, it can be seen that through the analysis of the reverse network pharmacology concept, the TCM components containing quercetin, epigallocatechin gallate, luteolin, fisetin, wogonin, triptolide, and the traditional Chinese medicines including Suberect Spatholobus Stem, Ginkgo Seed, Wild Buckwheat Rhizome, Sea Buckthorn Fruit, and Ginkgo Leafhave a very important role in the treatment of postmenopausal osteoporosis. Thus, this provides a direction for the in-depth research of targeted drugs for postmenopausal osteoporosis.

## Author contributions

**Conceptualization:** Daidai Wu.

**Data curation:** Daidai Wu.

**Formal analysis:** Daidai Wu.

**Funding acquisition:** Daidai Wu.

**Investigation:** Daidai Wu.

**Methodology:** Wanghuan Zhao, Yuehan Ni.

**Project administration:** Wanghuan Zhao, Yuehan Ni.

**Resources:** Wanghuan Zhao, Yuehan Ni.

**Software:** Daidai Wu.

**Supervision:** Wanghuan Zhao, Yuehan Ni.

**Validation:** Wanghuan Zhao, Yuehan Ni.

**Visualization:** Daidai Wu, Wanghuan Zhao, Yuehan Ni.

**Writing – original draft:** Daidai Wu.

**Writing – review & editing:** Daidai Wu.
